# Supplementary material for: Short Chain Fatty Acids and Fecal Microbiota Abundance in Humans with Obesity: A Systematic Review and Meta-Analysis
Source: Nutrients. 2019 Oct 18;11(10):2512. doi: 10.3390/nu11102512 (PMC6835694; doi:10.3390/nu11102512)

Supplemental Material

Supplementary Table S1: Search strategies

Supplementary Table S2: Reference list of papers excluded from the meta-analysis

Supplementary Table S3: Quality scoring for included five articles using Newcastle-Ottawa Scale (NOS) for case-control studies

Supplementary Table S4: Summary of studies included in the analysis of fecal microbiota levels in obese and nonobese subjects

Supplementary Table S5: Grade quality of evidence summary of the included studies

Supplementary Figure S1: Sensitivity analysis for the meta- analysis of short chain fatty acid and obesity

Supplementary Figure S2: Begg's Funnel plots with 95% confidence intervals for the meta-analysis of fecal microbiota

Supplementary Table S1. Search strategy

Common key words: "Short Chain Fatty Acid" and "Obesity" and "Human (not animal)"			
Keywords			
Subject	MeSH	Entry Terms	Text Words
Short Chain Fatty Acids AND Gut	"Fatty Acids, Volatile"[Mesh]	Volatile Fatty Acids<br>Fatty Acids, Short-Chain<br>Fatty Acids, Short Chain<br>Short-Chain Fatty Acids	
	"Gastrointestinal Microbiome"[Mesh]	Gastrointestinal Microbiomes<br>Microbiome, Gastrointestinal<br>Microbiomes, Gastrointestinal<br>Gut Microflora<br>Microflora, Gut<br>Gut Microbiota<br>Gut Microbiotas<br>Microbiota, Gut<br>Microbiotas, Gut<br>Gastrointestinal Flora<br>Flora, Gastrointestinal<br>Gut Flora<br>Flora, Gut<br>Gastrointestinal Microbiota<br>Gastrointestinal Microbiotas<br>Microbiota, Gastrointestinal<br>Microbiotas, Gastrointestinal<br>Gut Microbiome<br>Gut Microbiomes<br>Microbiome, Gut<br>Microbiomes, Gut<br>Gastrointestinal Microflora<br>Microflora, Gastrointestinal<br>Enteric Bacteria<br>Bacteria, Enteric<br>Intestinal Microbiome<br>Intestinal Microbiomes	Gut

		Microbiome, Intestinal<br>Microbiomes, Intestinal<br>Intestinal Microbiota<br>Intestinal Microbiotas<br>Microbiota, Intestinal<br>Microbiotas, Intestinal<br>Intestinal Microflora<br>Microflora, Intestinal<br>Intestinal Flora<br>Flora, Intestinal	
	"Obesity"[Mesh]		
	"Body Mass Index"[Mesh]	Index, Body Mass<br>Quetelet Index<br>Index, Quetelet<br>Quetelet's Index<br>Quetelets Index	
Database	Number	Search	Hits (numbers)
PubMed	#1	"Fatty Acids, Volatile"[Mesh]	96,222
	#2	"Fatty Acids, Volatile"[TW] OR "Volatile Fatty Acids"[TW] OR "Fatty Acids, Short-Chain"[TW] OR "Fatty Acids, Short Chain"[TW] OR "Short-Chain Fatty Acids"[TW]	10,872
	#3<br>Combine	("Fatty Acids, Volatile"[Mesh]) OR (("Fatty Acids, Volatile"[TW] OR "Volatile Fatty Acids"[TW] OR "Fatty Acids, Short-Chain"[TW] OR "Fatty Acids, Short Chain"[TW] OR "Short-Chain Fatty Acids"[TW]))	100,380
	#4	"Obesity"[Mesh]	184,859
	#5	"Obesity"[TW]	274,365
	#6	"Body Mass Index"[Mesh]	109,412
	#7	"Body Mass Index"[TW] OR "Index, Body Mass"[TW] OR "Quetelet Index"[TW] OR "Index, Quetelet"[TW] OR "Quetelet's Index"[TW] OR "Quetelets Index"[TW]	201,512
	#8<br>Combine	((("Obesity"[Mesh]) OR "Obesity"[TW]) OR "Body Mass Index"[Mesh]) OR (("Body Mass Index"[TW] OR "Index, Body Mass"[TW] OR "Quetelet Index"[TW] OR "Index, Quetelet"[TW] OR "Quetelet's Index"[TW] OR "Quetelets Index"[TW]))	402,828
	#9<br>Combine	((("Fatty Acids, Volatile"[Mesh]) OR (("Fatty Acids, Volatile"[TW] OR "Volatile Fatty Acids"[TW] OR "Fatty Acids, Short-Chain"[TW] OR "Fatty Acids, Short Chain"[TW] OR "Short-Chain Fatty Acids"[TW])))) AND (((("Obesity"[Mesh]) OR "Obesity"[TW]) OR "Body Mass Index"[Mesh]) OR (("Body Mass Index"[TW] OR "Index, Body Mass"[TW] OR "Quetelet Index"[TW] OR "Index, Quetelet"[TW] OR "Quetelet's Index"[TW] OR "Quetelets Index"[TW])))	1,200
	#10<br>Combine	#9 NOT ("Animals"[Mesh] NOT "Humans"[Mesh])	914
Database	Number	Search	Hits (numbers)
EMBASE	#1	Volatile fatty acid'/exp OR 'volatile fatty acid'	6,127
	#2	fatty acids, volatile'/exp OR 'fatty acids, volatile' OR 'volatile fatty acids' OR 'fatty acids, short-chain' OR 'fatty acids, short chain' OR 'short-chain fatty acids'	12,196
	#3<br>Combine	#1 OR #2	12,688
	#4	obesity'/exp OR 'obesity'	513,733

	#5	adipose tissue hyperplasia'/exp OR 'adipose tissue hyperplasia' OR 'adipositas'/exp OR 'adipositas' OR 'adiposity'/exp OR 'adiposity' OR 'alimentary obesity'/exp OR 'alimentary obesity' OR 'body weight, excess'/exp OR 'body weight, excess' OR 'fat overload syndrome'/exp OR 'fat overload syndrome' OR 'nutritional obesity'/exp OR 'nutritional obesity' OR 'obesitas'/exp OR 'obesitas' OR 'overweight'/exp OR 'overweight'	457,896
	#6	body mass'/exp OR 'body mass'	385,176
	#7	body mass index'/exp OR 'body mass index' OR 'index, body mass' OR 'quetelet index'/exp OR 'quetelet index' OR 'index, quetelet' OR 'quetelets index' OR 'bmi'/exp OR 'bmi' OR 'body ban mass'/exp OR 'body ban mass'	457,958
	#8 Combine	#4 OR #5 OR #6 OR #7	820,576
	#9 Combine	#3 AND #8	578
	#10 Combine	#9 NOT ('animal'/exp NOT 'human'/exp)	433
	#11 Combine	#9 NOT ('animal'/exp NOT 'human'/exp) AND [embase]/lim	397
Database	Number	Search	Hits (numbers)
Cochrane Library	#1	[mh "Fatty Acids, Volatile"]	3,536
	#2	"Fatty Acids, Volatile":ti,ab,kw OR "Volatile Fatty Acids":ti,ab,kw OR "Fatty Acids, Short-Chain":ti,ab,kw OR "Fatty Acids, Short Chain":ti,ab,kw OR "Short-Chain Fatty Acids":ti,ab,kw	371
	#3 Combine	#1 or #2	3,733
	#4	[mh Obesity]	11,184
	#5	Obesity:ti,ab,kw	23,363
	#6	[mh "Body Mass Index"]	9,225
	#7	"Body Mass Index":ti,ab,kw OR "Index, Body Mass":ti,ab,kw OR "Quetelet Index":ti,ab,kw OR "Index, Quetelet":ti,ab,kw OR "Quetelet's Index":ti,ab,kw OR "Quetelets Index":ti,ab,kw	23,605
	#8 Combine	{or #4-#7}	38,923
	#9 Combine	#3 and #8	164
	#10 Combine	#9 not ([mh Animals] not [mh Humans])	164

Supplementary Table S2. Reference list of papers excluded from the meta-analysis

(A) Not relevant to the exposure and outcome: short chain fatty acids and obesity: (1-8)

(B) Review: (9-18)

(C) Studies without sufficient information on short chain fatty acid levels and obesity, including the mean, median, standard deviation or standard error. (19-22)

1. Canfora EE, van der Beek CM, Jocken JWE, et al. Colonic infusions of short-chain fatty acid mixtures promote energy metabolism in overweight/obese men: a randomized crossover trial. Sci Rep 2017;7:2360.
2. Duncan SH, Belenguer A, Holtrop G, et al. Reduced dietary intake of carbohydrates by obese subjects results in decreased concentrations of butyrate and butyrate-producing bacteria in

feces. Appl Environ Microbiol 2007;73:1073-8.
3. Ferchaud-Roucher V, Pouteau E, Piloquet H, et al. Colonic fermentation from lactulose inhibits lipolysis in overweight subjects. Am J Physiol Endocrinol Metab 2005;289:E716-20.
4. Koliada A, Syzenko G, Moseiko V, et al. Association between body mass index and Firmicutes/Bacteroidetes ratio in an adult Ukrainian population. BMC Microbiol 2017;17:120.
5. Layden BT, Yalamanchi SK, Wolever TM, et al. Negative association of acetate with visceral adipose tissue and insulin levels. Diabetes Metab Syndr Obes 2012;5:49-55.
6. Lin HV, Frassetto A, Kowalik EJ, Jr., et al. Butyrate and propionate protect against diet-induced obesity and regulate gut hormones via free fatty acid receptor 3-independent mechanisms. PLoS One 2012;7:e35240.
7. Salonen A, Lahti L, Salojarvi J, et al. Impact of diet and individual variation on intestinal microbiota composition and fermentation products in obese men. Isme j 2014;8:2218-30.
8. Sanna S, van Zuydam NR, Mahajan A, et al. Causal relationships among the gut microbiome, short-chain fatty acids and metabolic diseases. Nat Genet 2019;51:600-605.
9. Arora T, Sharma R, Frost G. Propionate. Anti-obesity and satiety enhancing factor? Appetite 2011;56:511-5.
10. Chambers ES, Preston T, Frost G, et al. Role of Gut Microbiota-Generated Short-Chain Fatty Acids in Metabolic and Cardiovascular Health. Curr Nutr Rep 2018;7:198-206.
11. den Besten G, van Eunen K, Groen AK, et al. The role of short-chain fatty acids in the interplay between diet, gut microbiota, and host energy metabolism. J Lipid Res 2013;54:2325-40.
12. Kasubuchi M, Hasegawa S, Hiramatsu T, et al. Dietary gut microbial metabolites, short-chain fatty acids, and host metabolic regulation. Nutrients 2015;7:2839-49.
13. Li X, Shimizu Y, Kimura I. Gut microbial metabolite short-chain fatty acids and obesity. Biosci Microbiota Food Health 2017;36:135-140.
14. McNabney SM, Henagan TM. Short Chain Fatty Acids in the Colon and Peripheral Tissues: A Focus on Butyrate, Colon Cancer, Obesity and Insulin Resistance. Nutrients 2017;9.
15. Morrison DJ, Preston T. Formation of short chain fatty acids by the gut microbiota and their impact on human metabolism. Gut Microbes 2016;7:189-200.
16. Murugesan S, Nirmalkar K, Hoyo-Vadillo C, et al. Gut microbiome production of short-chain fatty acids and obesity in children. Eur J Clin Microbiol Infect Dis 2018;37:621-625.
17. Prasad KN, Bondy SC. Dietary Fibers and Their Fermented Short-Chain Fatty Acids in Prevention of Human Diseases. Mech Ageing Dev 2018.
18. Topping DL, Clifton PM. Short-chain fatty acids and human colonic function: roles of resistant starch and nonstarch polysaccharides. Physiol Rev 2001;81:1031-64.
19. de la Cuesta-Zuluaga J, Mueller NT, Alvarez-Quintero R, et al. Higher Fecal Short-Chain Fatty Acid Levels Are Associated with Gut Microbiome Dysbiosis, Obesity, Hypertension and Cardiometabolic Disease Risk Factors. Nutrients 2018;11.

20. Goffredo M, Mass K, Parks EJ, et al. Role of Gut Microbiota and Short Chain Fatty Acids in Modulating Energy Harvest and Fat Partitioning in Youth. J Clin Endocrinol Metab 2016;101:4367-4376.
21. Murugesan S, Ulloa-Martinez M, Martinez-Rojano H, et al. Study of the diversity and short-chain fatty acids production by the bacterial community in overweight and obese Mexican children. Eur J Clin Microbiol Infect Dis 2015;34:1337-46.
22. Teixeira TF, Grzeskowiak L, Franceschini SC, et al. Higher level of faecal SCFA in women correlates with metabolic syndrome risk factors. Br J Nutr 2013;109:914-9.

Supplementary Table S3. Quality scoring for included five articles using Newcastle-Ottawa Scale (NOS) for case-control studies

Quality scoring for studies included in the meta-analysis. For each paper total 'star' (★) score was given consisting of; a letter (a, b or c) that stands for which of the NOS quality coding item list describe the paper more.

Reference	Case definition	Case representation	Control selection	Control definition	Comparability	Exposure ascertainment	Same method of ascertainment	Response rate	Total ★ scores
Barczyńska, 2018	a★	a★	c0	a★	a★	a★	a★	a★	7
Dugas, 2018	a★	a★	a★	a★	a★	a★b★	a★	a★	9
Riva, 2017	a★	a★	b0	a★	a★	a★b★	a★	a★	8
Fernandes, 2014	a★	a★	a★	a★	a★	a★	a★	a★	8
Rahat-Rozenbloom, 2014	a★	a★	a★	a★	a★	a★b★	a★	a★	9
Schwiertz, 2010	a★	a★	a★	a★	a★	a★	a★	a★	8
Todesco, 1993	a★	a★	c0	a★	a★	a★	a★	a★	7

NOS coding manual for case-control studies

Selection

1) Is the Case Definition Adequate?

a) Yes, with independent validation ★ (e.g. >1 person/record/time/process to extract information, or reference to primary record source such as x-rays or

medical/hospital records)

b) Yes, record linkage (e.g. ICD codes in database) or self-report with no reference to primary record

c) No description

2) Representativeness of the Cases

a) Consecutive or obviously representative series of cases ⋆

b) Potential for selection biases or not stated

3) Selection of Controls

a) Community controls ⋆ (i.e. same community as cases and would be cases if had outcome)

b) Hospital controls, within same community as cases (i.e. not another city) but derived from a hospitalized population

c) No description

4) Definition of Controls

a) No history of disease (endpoint) ⋆

b) No description of source

Comparability

1) Comparability of cases and controls on the basis of the design or analysis

A maximum of 2 stars can be allotted in this category

a) Study controls for the most important factor ⋆

b) Study controls for any additional factor ⋆ (This criteria could be modified to indicate specific control for a second important factor.)

Exposure

1) Ascertainment of exposure

a) Secure record (eg surgical records) ★

b) Structured interview where blind to case/control status ★

c) Interview not blinded to case/control status

d) Written self–report or medical record only

e) No description

2) Same method of ascertainment for cases and controls

a) Yes ★

b) No

3) Non-Response Rate

a) Same rate for both groups ★

b) Non respondents described

c) Rate different and no designation

Supplementary Table S4 Summary of studies included in the analysis of fecal microbiota levels in obese and nonobese subjects

BMI category	Phylum	Microbiota	Unit	Obese			Nonobese		
Study				N	Mean	SD*	N	Mean	SD
BMI-Z score				>0.3			<0.3		
Riva et al, 2017	Bacteroidetes	No available	[a]Percent (%)	42	16.6	11.8	36	30	12.6
Riva et al, 2017	Firmicutes	No available	[a]Percent (%)	42	72.1	12.1	36	60.9	14.1

BMI (kg/m^2)				>25			<25		
Fernandes et al, 2014	Firmicutes	Clostridium coccoides	log cells/g	42	9.2	0.65*	52	9.2	0.72*
Fernandes et al, 2014	Firmicutes	Clostridium leptum	log cells/g	42	9.5	0.65*	52	9.6	0.72*
Fernandes et al, 2014	Bacteroidetes	Bacteroides/Prevotella	log cells/g	42	8.2	1.3*	52	8.6	0.72*
Rahat-Rozenbloom et al, 2014	Firmicutes	Not available	[a]Percent (%)	11	83.1	13.6*	11	69.5	19.24*
Rahat-Rozenbloom et al, 2014	Bacteroidetes	Not available	[a]Percent (%)	11	6.4	14.26*	11	19.4	20.23*
Schwiertz et al, 2010	Firmicutes	Clostridium leptum	log cells/g	35	10.3	0.64	30	10.4	0.35
Schwiertz et al, 2010	Firmicutes	Clostridium coccoides	log cells/g	35	10.3	0.63	30	10.3	0.34
Schwiertz et al, 2010	Firmicutes	Lactobacillus/Enterococcus	log cells/g	35	6.8	0.64	30	7	0.53
Schwiertz et al, 2010	Firmicutes	E. cylindroides	log cells/g	35	6.9	1.65	30	7.7	1.24
Schwiertz et al, 2010	Firmicutes	Veillonella	log cells/g	35	6.4	0.83	30	6.5	0.59
BMI (kg/m^2)				>30			<25		
Schwiertz et al, 2010	Firmicutes	Clostridium leptum	log cells/g	30	10.2	0.71	30	10.4	0.35
Schwiertz et al, 2010	Firmicutes	Clostridium coccoides	log cells/g	30	10	0.5	30	10.3	0.34
Schwiertz et al, 2010	Firmicutes	Lactobacillus/Enterococcus	log cells/g	30	6.9	0.45	30	7	0.53
Schwiertz et al, 2010	Firmicutes	E. cylindroides	log cells/g	30	7.7	1.85	30	7.7	1.24
Schwiertz et al, 2010	Firmicutes	Veillonella	log cells/g	30	6.3	1.44	30	6.5	0.59
BMI (kg/m^2)				>25			<25		
Schwiertz et al, 2010	Bacteroidetes	Bacteroides	log cells/g	35	10.4	0.49	30	10.2	0.47
Schwiertz et al, 2010	Bacteroidetes	Prevotella	log cells/g	35	5.9	2	30	6.6	1.42
BMI (kg/m^2)				>30			<25		
Schwiertz et al, 2010	Bacteroidetes	Bacteroides	log cells/g	30	10.2	0.74	30	10.2	0.47
Schwiertz et al, 2010	Bacteroidetes	Prevotella	log cells/g	30	5.9	2.01	30	6.6	1.42

[a] percent OTU/total OTU in feces; Calculated SD*=SE× $\sqrt{\text{sample size}}$; BMI, body mass index; OTU, Operational taxonomic unit; SD, standard deviation; SE, standard error

Supplementary Table S5. Grade quality of evidence summary of the included studies

Quality assessment							Summary of findings					Importance
							No. of patients		Effect		Quality	
No. of studies	Design	Risk of bias	Inconsistency	Indirectness	Imprecision	Other considerations	Cases	Control	SMD (95% CI)	[a]Absolute (95% CI)		
**Total SCFA increase in obese individuals**												

6	Case-control study	Serious	Serious	Not serious	Serious	No publication bias suspected	241/462 (52.2%)	221/462 (47.8%)	0.54 (-0.34 to 1.4
**Acetate increase in obese individuals**									
6	Case-control study	Serious	Serious	Not serious	Serious	No publication bias suspected	229/437 (52.4%)	208/437 (47.6%)	0.87 (0.24 to 1.5
**Propionate increase in obese individuals**									
5	Case-control study	Serious	Serious	Not serious	Serious	No publication bias suspected	221/422 (52.4%)	201/422 (47.6%)	0.86 (0.35 to 1.3
**Butyrate increase in obese individuals**									
5	Case-control study	Serious	Serious	Not serious	Serious	No publication bias suspected	221/402 (52.4%)	201/422 (47.6%)	0.78 (0.29/1.27
**Fecal Firmicutes abundant in obese individuals**									
5	Case-control study	Serious	Serious	Not serious	Not serious	No publication bias suspected	462/913(49.4%)	451/913 (50.6%)	-0.10 (-0.31/0.10
**Fecal Bacteroidetes abundance in obese individuals**									
4	Case-control study	Serious	Serious	Not serious s	Serious	No publication bias suspected	225/442 (50.6%)	219/442 (49.3%)	-0.36 (-0.73/0.01

CI, confidence interval; SD, standard deviation; SMD, standardized mean difference; SCFA, short chain fatty acid. [a]The rules of effect sizes (An SMD below 0.5 was considered small, 0.5–0.8 was considered moderate, and over 0.8 was considered la

Supplementary Figure S1. Sensitivity analysis for the meta- analysis of short chain fatty acid and obesity; a, study-specific combined SMDs; b, the sensitivity analysis excluded the study by Barczyńska et al. and the study by Fernandes et al.

a

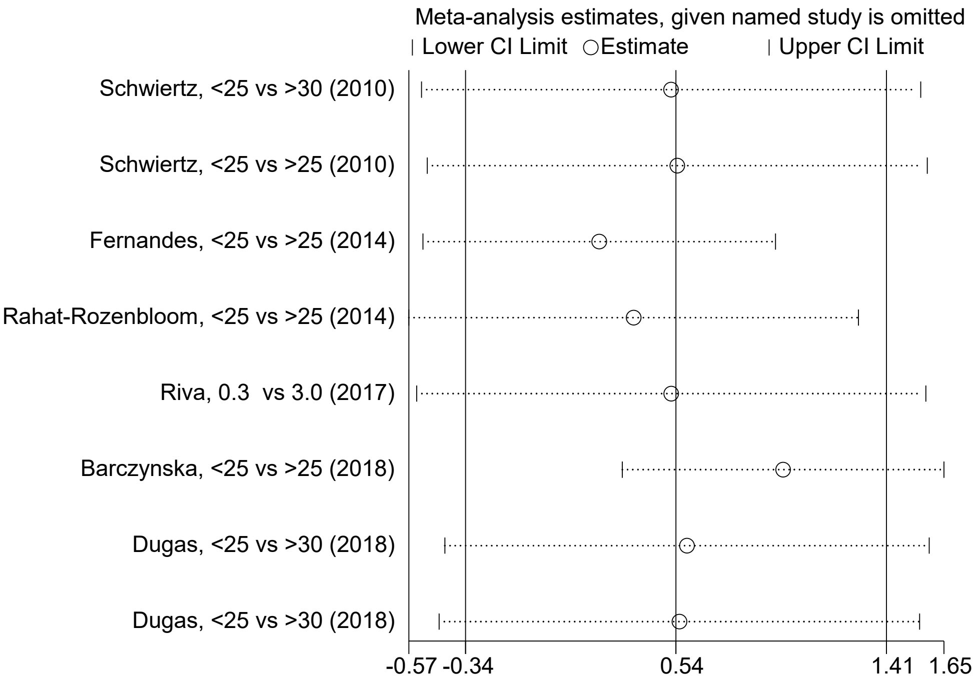

b

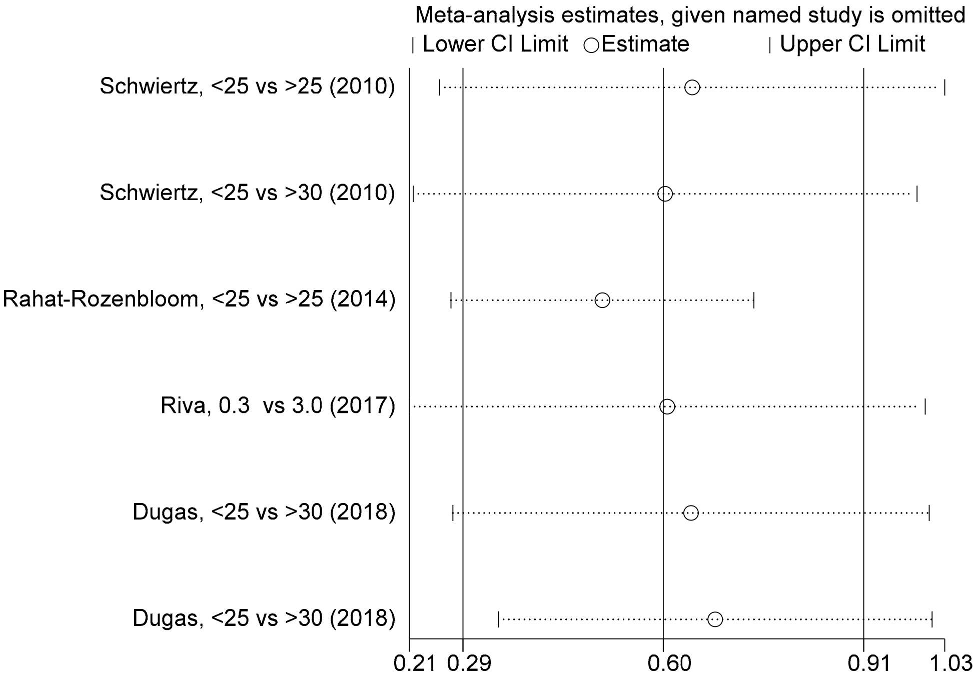

Supplementary Figure S2. Begg’s Funnel plots with 95% confidence intervals for the meta-analysis of fecal microbiota. a. Bacteroidetes, b. Firmicutes

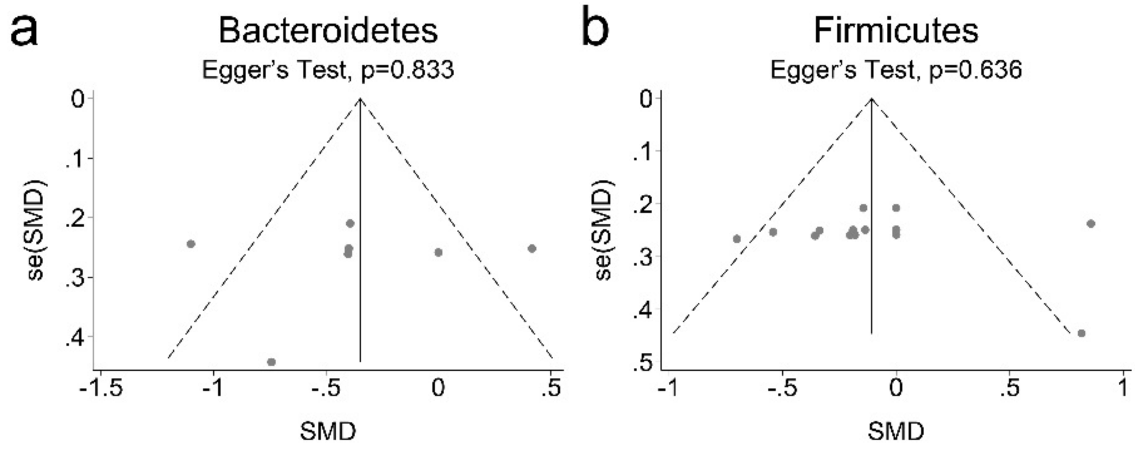

Supplement: Supplementary file 1 [file nutrients-11-02512-s001.pdf]
